# Cognitive Impairment and Brain Reorganization in MS: Underlying Mechanisms and the Role of Neurorehabilitation

**DOI:** 10.3389/fneur.2020.00147

**Published:** 2020-03-06

**Authors:** Grigorios Nasios, Christos Bakirtzis, Lambros Messinis

**Affiliations:** ^1^Department of Speech and Language Therapy, School of Health Sciences, University of Ioannina, Ioannina, Greece; ^2^Department of Neurology, The Multiple Sclerosis Center, AHEPA University Hospital, Aristotle University of Thessaloniki, Thessaloniki, Greece; ^3^Neuropsychology Section, Departments of Neurology and Psychiatry, University of Patras Medical School, Patras, Greece

**Keywords:** multiple sclerosis, cognitive impairment, brain reorganization, cognitive rehabilitation, neuromodulation

## Abstract

Multiple sclerosis (MS) is a chronic, immune-mediated, inflammatory, and degenerative disease of the central nervous system (CNS) that affects both white and gray matter. Various mechanisms throughout its course, mainly regarding gray matter lesions and brain atrophy, result in cognitive network dysfunction and can cause clinically significant cognitive impairment in roughly half the persons living with MS. Altered cognition is responsible for many negative aspects of patients' lives, independently of physical disability, such as higher unemployment and divorce rates, reduced social activities, and an overall decrease in quality of life. Despite its devastating impact it is not included in clinical ratings and decision making in the way it should be. It is interesting that only half the persons with MS exhibit cognitive dysfunction, as this implies that the other half remain cognitively intact. It appears that a dynamic balance between brain destruction and brain reorganization is taking place. This balance acts in favor of keeping brain systems functioning effectively, but this is not so in all cases, and the effect does not last forever. When these systems collapse, functional brain reorganization is not effective anymore, and clinically apparent impairments are evident. It is therefore important to reveal which factors could make provision for the subpopulation of patients in whom cognitive impairment occurs. Even if we manage to detect this subpopulation earlier, effective pharmaceutical treatments will still be lacking. Nevertheless, recent evidence shows that cognitive rehabilitation and neuromodulation, using non-invasive techniques such as transcranial magnetic or direct current stimulation, could be effective in cognitively impaired patients with MS. In this Mini Review, we discuss the mechanisms underlying cognitive impairment in MS. We also focus on mechanisms of reorganization of cognitive networks, which occur throughout the disease course. Finally, we review theoretical and practical issues of neurorehabilitation and neuromodulation for cognition in MS as well as factors that influence them and prevent them from being widely applied in clinical settings.

## Introduction

People living with Multiple sclerosis (pwMS) commonly exhibit cognitive deficits, which negatively affects them multidimensionally (1). Daily functioning, decision making, vocational activities, marital status, socialization, behavior, mood, balance and mobility, and compliance with medications can be affected. The medical community, due to their often subtle nature and the difficulty that exists in detecting these deficits during routine clinical practice, was initially slow to appreciate them as a core clinical symptom of MS. We recently proposed a practical algorithm for clinicians regarding “what,” “why,” “how,” and “when” to measure (2). Today, most of the evidence suggests that cognitive impairment in MS patients is present during all disease stages and across all disease clinical subtypes (3–5). CI can be detected even prior to diagnosis (6) in radiologically isolated syndrome (RIS) (7), clinically isolated syndrome (CIS) (8), “benign” MS (9), and the pediatric MS population (10). Deficits appear to be more frequent and widespread in the progressive rather than the relapsing form of the disease (11–13). Even then, roughly half of the patients do not exhibit prominent CI. CI is also linked to disease duration (12, 14), tissue damage and atrophy (15–17), and cognitive network efficiency (18, 19). Some cognitive domains appear to be more commonly compromised than others: information processing efficiency, episodic memory, attention, and executive functioning are found predominantly to be detrimentally affected in MS (20, 21). Among these domains the most common pattern involves circumscribed deficits as a combination of one or two of the abovementioned domains (e.g., attention/processing speed, learning/ memory, and or executive functions).

On the other hand, social cognitive deficits are an underestimated but important aspect of impairment in MS, reflecting how people process, store, and apply information in social interactions. Deficits in these domains have been associated with reduced quality of life, even after controlling for severity and duration of the disease, age, and neurocognitive performance (22, 23). This type of impairment is not entirely dependent on and parallel to general cognitive dysfunction—some patients experience disorganization in their social life before significant or detectable cognitive impairment is evident. The decrease in performance of social cognition (SC) tasks may reflect changes in brain activity and brain structure, either general or regional (22, 24).

In order to answer the question of why half of pwMS do not exhibit CI, approaching the disease within the context of its trilateral interference of tissue damage, tissue repair, and brain reorganization (25) may be helpful: tissue damage is indeed a matter of time and disease type and severity, and it can be partially influenced by the early introduction of efficacious disease modifying therapies (DMTs). Tissue repair is served by various mechanisms that are not yet well-illuminated, may vary in affected individuals, is altered by factors such as co-morbidity, stress, or lifestyle, and, unfortunately, was not targeted with specific medications until now; functional brain reorganization, in other words neuroplasticity, is the intrinsic force fighting the consequences of disease progression and can hopefully be managed through neurorehabilitation interventions.

In the following sections we have addressed issues concerning CI and functional brain reorganization in MS; we focused on cognitive network alterations, efficiency, and collapse, the role of inflammation, and mechanisms underlying synaptopathy and synaptogenesis. We further discussed the potential role of cognitive rehabilitation and neuromodulation in retaining and enhancing network efficiency in a clinically meaningful way.

### Networks-Connectivity-Brain Reorganization

Brain reorganization in MS is studied intensively by mainly functional neuro-imaging methods. Functional connectivity (FC) at rest and during tasks can detect both hyperconnectivity and hypoconnectivity in brain networks. This can compensate for tissue damage, allowing pwMS to adequately cope with everyday cognitive tasks despite continuing structural brain damage. These alterations can be adaptive or maladaptive. We recently summarized the basic concepts, and limitations, of functional brain reorganization in MS (26). Even from early disease phases—in patients with CIS—dynamic changes in functional brain networks have been observed, resulting in the maintenance of normal efficiency in the brain and consequently representing a compensatory effect (27). A mixed pattern of hypoactivity and hyperactivity was found, by means of rs-fMRI, in pwMS at different stages of disease. Relapsing Remitting Multiple Sclerosis (RRMS) individuals with short disease duration, and RRMS with similar disabilities but longer disease duration, were characterized by a clearly distinct pattern of FC that involved predominantly sensory and cognitive networks, respectively (28). In a longitudinal 1-year network connectivity study, measures were compared between early RRMS patients and healthy matched controls as well as between patients with and without disease activity (29). The study reported that the strengthening of local network properties was only detectable in the cortex of patients and occurred independently of their disease activity. Authors discuss these changes as an adaptive mechanism that is important for maintaining brain function in response to neuroinflammation. In another study, patients who converted to MS exhibited significantly greater network connectivity at baseline than non-converters (30). Cader et al. concluded that both forms of adaptive functional change—that is, the enhancement of interactions between brain regions normally recruited, and the recruitment of alternative areas, or the use of complementary cognitive strategies—could limit clinical expression of the disease, particularly that of CI (31). As a rule, PwMS perform better in cognitive tasks if they have preserved fMRI activity of their frontal lobes (32). Not only functional but also structural connectivity matters. Llufriu et al. investigating reorganization mechanisms at the structural level that are related to attention and executive performance in pwMS, and they found that the right pallidum and left insula within the frame of the brain's reorganization functioned as hubs in patients (33). However, we must keep in mind that several limitations exist that relate to the role of altered connectivity throughout the disease; it is still questionable whether the observed changes are relevant to cognitive performance and whether or not they are adaptive (26). As the disease progresses, network efficiency is challenged by tissue damage, restorative mechanisms become inadequate, and, finally, the network collapses (18).

### Regional Tissue Damage and Atrophy

Gray matter (GM) lesions (15) and GM atrophy (16) play an important role in CI. Across the disease span, if left untreated, the white matter atrophy rate remains rather stable at 3-fold normal, but GM atrophy rate dramatically increases from 3.4-fold normal in CIS to 14-fold normal in SPMS (17). This localized GM atrophy has recently been found to be regionally selective, mainly involving deep structures, such as the thalamus, putamen, and caudate, and cortical regions, such as the sensorimotor cortex, insula, superior temporal, and cingulate gyrus, while these regions were functionally connected (34). In a 5-years follow-up study, it was shown that structural damage and especially cortical atrophy may predict cognitive decline in PwMS (35). Among strategic GM structures, the thalamus, basal ganglia, and hippocampus seem to play a central role. Thalamic volume declines faster in pwMS throughout the disease, and it was proposed to serve as a biomarker of degeneration (36). Its volume, shape, and function are related to cognitive performance in MS (37–39). In early RRMS patients (duration of disease <3 years), CI was detected in 28% over a 2-years follow up period, and in this subgroup a significant reduction in the percentage of thalamus volume was observed compared with the cognitively intact group (40). In a large cohort of MS patients, with various forms and stages of the disease, Rocca et al. investigated rs-FC abnormalities within the principal brain networks in PwMS (41). They found a complex pattern of decreased and increased rs-FC at a regional level: reduced thalamic rs-FC correlated with better neuropsychological performance, whereas, for all the remaining networks, reduced FC correlated with more severe clinical/cognitive impairment. This finding was in line with the observation of Zhou et al. who found that increased thalamic intrinsic oscillation amplitude in RRMS patients was associated with slowed cognitive processing, representing ineffective reorganization (42). Resting-state magneto-encephalography recordings from pwMS and healthy controls offered similar evidence, illustrating “the relationship between thalamic atrophy, altered functional connectivity and clinical and cognitive dysfunction in MS” (43). The importance of thalamic involvement in disease progression and CI is highlighted by Minagar et al. (44), who recommended that thalamic volume should be utilized as a biomarker in MS clinical trials. In a recent study where neuropsychological and MRI data of 375 PwMS were analyzed, altered performance on neuropsychological tests assessing attention and executive function was associated with caudate volume and posterior cingulate/precuneus atrophy, while tests primarily evaluating memory strongly correlated with thalamic volume (45). In untreated CIS patients, load-dependent dysfunction of the putamen was related to impaired performance during attention tasks (46). Amygdala atrophy was found to be the main predictor of impairment of social cognition (SC) in PwMS (24). In agreement with this, Pitteri at al. correlated bilateral amygdala damage, as measured by cortical lesion volume (CLV), to affected SC in PwMS, even in the absence of CI (47). In a multicenter study of structural correlates of CI in MS, the best predictors of CI were found to be atrophy of the hippocampus and deep GM nuclei (48). The importance of structural and functional integrity of the hippocampus was highlighted by Sumowski et al. (49), who investigated the neural basis of reserve against memory decline in PwMS, linking greater intellectual enrichment and better memory to larger hippocampal volume and supporting the argument that larger hippocampal volume is a key component of reserve against memory decline in MS. The hippocampus of PwMS usually has a high lesion load, demyelination, neuronal damage, synaptic dysfunction, neurotransmitter level reduction, and disconnection, linking hippocampus pathology not only to CI but also to the reorganization capacity of broader networks (48, 50–55). A biomarker indicating deep GM structures, especially thalamus and hippocampus volume and status, could ideally give provision of the cognitive status, insights for reorganization dynamics, and cues for therapeutic decisions.

### Synaptopathy in MS

We formerly recognize MS as a myelin-targeting autoimmune disease of the CNS, causing inflammation, white (and gray) matter tissue damage, and neurodegeneration, but the influence of GM pathology was recognized later. Loss and malfunction of synapses could offer an explanation for this role. Recent clinical and experimental studies link inflammation to neurodegeneration, illuminating the contribution not only of visible structural damage but also of synaptic dysfunction in the pathophysiology of both motor and cognitive functions in MS. Many studies have provided robust evidence for diffuse synaptic dysfunction being present in both MS and EAE (experimental autoimmune encephalomyelitis, the animal model of MS) throughout the disease course (56–59). Stampanoni Bassi et al. and Mandolesi et al. have discussed thoroughly the molecular and cellular mechanisms underlying alterations in synaptic function and structure (58, 59). They emphasized the role of inflammation in neurotransmitters' imbalance; increased glutamate-mediated and reduced GABA-mediated signaling along with the excitotoxic effects of increased glutamate levels in the synaptic cleft may lead to synaptic degeneration, which, interestingly, may occur independently of GM demyelination and neuronal loss. Of course, synaptopathy can also be the consequence of axonal damage, but it is present from the initial phase of the disease when one could not yet expect that much of axonal damage. This supports the idea that synaptopathy, rather than axonal loss, leads to accumulation of disability, at least early in the disease course. Focusing our attention to the synaptic level, we must keep in mind the role of long-term potentiation (LTP) and long-term depression (LTD) which represent core underlying mechanisms of synaptic plasticity, i.e., maintaining synaptic strengths, efficacy, and stability, adjusted dynamically by neural activity. The ways structural and functional damage result in synaptic failure, network dysfunction, and, therefore, CI have recently been reviewed by Di Filippo et al. (60).

Fortunately, and unlike the loss of neurons, the loss of synapses is reversible. New synapses can be generated, and dysfunctional synapses can be repaired, resulting in restoration of functions or even reversing the progression of the disease, as has been shown both in EAE animals (61) and in pwMS (62). Targeting synapses therapeutically can be achieved by at least some of the DMTs, especially those that pass the blood–brain barrier, by reducing inflammation and tissue damage or even by exerting direct neuroprotective effects (59). Since MS-related disability progression can be modulated by plasticity, and plasticity can be enhanced by neurorehabilitation and neuromodulation, these latter approaches could be therapeutically used to delay progression; this promotes brain reorganization, mainly at the synaptic level, since their mechanisms of action include LTP/LTD.

### Neurorehabilitation and Neuromodulation for MS-Related Cognitive Impairment

Three decades have passed since the first published reports under the search items “cognitive rehabilitation” and “MS” appeared in Pubmed (1,086 research items in total, two in year 1990, 160 in 2019, page visited on 1.1.2020). In 1993, DeLuca and Johnson stated that, since cognitive dysfunction negatively impacts the lives of pwMS, it must be targeted by neurorehabilitation (63). They described the complicated landscape of cognitive rehabilitation (CR) in MS since, due to “the heterogeneous nature of the CNS lesions, each person with MS brings a unique pattern of cognitive difficulties,” and, furthermore, “effective CR in MS goes beyond simple assessment and treatment of specific deficits” (63). Since then, more questions than answers have arisen. Questions regarding the evaluation of CI and the type of CR should be investigated (64); evidence and methodological restrictions of CR protocols (1, 65–68) as well as many practical issues of CR, such as the mechanisms of action, duration, intensity, frequency, repeatability, consistency and duration of effects, ecological validity, and “the transportability of such interventions under real-world conditions,” (69) should be fully explored. There is another major practical restriction: in most countries, there is lack of providers (clinical neuropsychologists and trained speech language therapists) able to apply these methods (64). These restrictions are reflected in the low rate of pwMS exposed to CI, even in countries, such as Finland, with high incidences of MS and advanced health services (70); pharmacological treatments for MS-related CI are still lacking (64, 69, 71). In order to move faster from the research fields to clinical grounds and offer CR as standard-of-care treatment, a roadmap was recently proposed by Sandroff and DeLuca (69). One major point concerning CR for MS-related CI is its mechanism of action, which seems to be the enhancement of neuroplasticity. Prosperini et al. reviewed the literature, showing that both motor and cognitive rehabilitation enhance functional and structural brain plasticity in pwMS, and this enhancement is specifically linked to the trained domain (72). Recently, Prosperini and Di Filippo updated evidence from animal models and pwMS on plasticity following rehabilitation (73).

Neuromodulation is technology acting directly upon the nervous system. Non-invasive brain stimulation (NIBS) refers to the application on the scalp of a changing magnetic field [transcranial magnetic stimulation, TMS; (74)], or low-intensity electrical current [transcranial direct current stimulation, tDCS; (75)] over a short period of time, both of which are methods capable of altering brain function since they have cumulative and long lasting effects. The reader is referred to relevant, recently-published reviews for the use of rTMS (26) and tDCS (76) in the management of MS-related symptoms. Both techniques are easily applicable, affordable, and rather safe, with tDCS being much cheaper, able to be self-administered at home by remote supervision (77) and, more importantly, while performing a task (“on-line”), while rTMS must be carried out in the presence of a skilled clinician and during rest (“off-line”). Changing stimulus parameters and/or electrode polarity, excitation (high-frequency rTMS or anodal tDCS), or inhibition protocols (low-frequency rTMS or cathodal tDCS) can be designed, inducing LTP-like and LTD-like plasticity, respectively; this also influences brain plastic changes, acting therapeutically, either alone or in combination with CR and/or exercise. The neurobiological basis and the effectiveness of NIBS for MS-related symptoms have been updated by Leocani et al. (78). Among the issues are the differences from patient to patient in the electrical current flow induced by NIBS techniques; these depend on the volume and topography of the lesions, and different patients may need different NIBS protocols. However, since the use of tDCS during cognitive rehabilitation may improve outcomes and provide beneficial results in a short time (79), newer large-scale studies should further be performed in order to provide robust evidence to support the implementation of NIBS in routine practice (80).

## Discussion

Here, we have discussed the mechanisms underlying cognitive impairment and the reorganization of cognitive networks in MS and issues for the implementation of neurorehabilitation and neuromodulation in clinical settings. Recently, Harel et al. presented “the bright side” of cognitive function in multiple sclerosis (81). Indeed, nearly 20 years after the disease onset, more than three out of 10 the pwMS of their large sample were cognitively intact. But there is also a “dark side”: two out of 10 were seriously cognitively handicapped, while one of 10 were both severely affected cognitively and physically. Furthermore, the disease does not last only 20 years, but it is lifelong; the mean age of pwMS in this study was noted as 49.3 years, meaning that the majority of them will still be alive 10–20 years later, the proportion of disabled will definitely increase, as will co-morbidities (82), and their treatment opportunities will be narrowed. Unfortunately, as it has been shown in the EAE model of MS, the loss of synapses can occur early in the disease course, irrespectively of demeylination (83). Also, neurodegeneration and resulted atrophy is proven to be evident, subclinically, even from the radiologically isolated syndrome (84). These two mechanisms could explain why CI can be present even before the time of diagnosis. Additionally, what we have undoubtedly learned is that CI at diagnosis predicts worse future disease progression (85), impairment of specific cognitive sub-domains might better predict progression (86), and patients with pediatric onset MS are more likely to have CI than patients with disease onset in adulthood, independent of age, or disease duration (87). A patient with less severe tissue damage, less atrophy, spared key brain loci, more effective tissue repair mechanisms, and enhanced brain reorganization capacities could remain cognitively intact, even decades after disease onset, and vice versa. In the first instance, the patient will probably constitute “the bright side” of the ~50% cognitively intact pwMS. What about the other 50%? For them, more than the others, “time is brain,” and delays in appropriate clinical decisions will probably cost their transition to the “dark side” of CI. In our opinion, early clinical detection of CI, coupled with evidence of structural damage in key brain regions (thalamus, hippocampus, amygdala, etc.) and altered network connectivity, could serve toward this goal. Therefore, there is an urgent need to identify this high-risk subpopulation of pwMS, and, since it is not possible to be achieved early in the disease course through clinical and conventional neuroimaging grounds only (88), we have to find biomarkers (molecular, metabolic, imaging, and clinical) to detect earlier CNS pathology “before structural tissue damage has become definite” (89). Impaired cognition is associated with early increases in FC, which then decreases due to the exhaustion of compensating mechanisms, forming the “inverted U” rs-FC curve (89). Indeed, patients who converted to MS exhibited “significantly greater network connectivity at baseline than non-converters” and a “subsequent connectivity loss over time, not observed in the non-converters' network” (30, 90). Therefore, despite methodological difficulties (91), widely available imaging markers could soon offer more (92). As we have previously stated, a biomarker composed of deep GM structures, and especially the thalamus and hippocampus volume and status, could “reflect” the cognitive status and provide insight into reorganization dynamics. Once recognized, this subpopulation should be treated more aggressively with highly efficacious DMTs and, ideally, neurorehabilitation and neuromodulation procedures.

We are not optimistic about the introduction of CR and NBIS in routine clinical care, at least in the near future, and there are reasons for this: neurorehabilitation is ultimately “treatment of the whole person” (69) and should, in other words, be tailored to every individual person. This means that it is almost impossible to include parameters of every pwMS—as they all have distinct disease characteristics, personalities, and life variables—into clinical trials and then translate their results back to a highly individualized procedure, maximizing the possibilities to identify the right patient and carry out the appropriate treatment. Moreover, as we discussed, there are many unsolved practical issues for their implementation in clinical practice. For NIBS techniques, additional issues arise, including identifying sites for brain stimulation, depending on brain lesion topography of every single pwMS, and simultaneously choosing excitatory or inhibitory protocols, or combination of both. These sophisticated individualized treatments must be carried out by a large number of trained clinicians, who do not yet exist. Finally, the (large) financial cost must be covered somehow.

What is feasible? Diagnose MS sooner, detect CI earlier and include it in clinical decisions, start treatment early and constantly follow through with more effective DMTs, find medications for CNS tissue repair, adopt strategies for “reserve and brain maintenance” (93), and, of course, do more research on neurorehabilitation and neuromodulation since they seem to be at least in part effective, even in advanced disease stages (94), and may enhance the brain's plasticity and alter disease course. This is all with the ultimate goal of implementing these methods in routine MS management.

## Author Contributions

GN conceptualized this mini-review article. GN, CB, and LM wrote the first draft of the manuscript. All authors were involved in the critical reading and the revision process.

### Conflict of Interest

The authors declare that the research was conducted in the absence of any commercial or financial relationships that could be construed as a potential conflict of interest.

## Abbreviations

CI: Cognitive impairment
CIS: clinically isolated syndrome
CNS: Central nervous system
CR: cognitive rehabilitation
DMTs: disease modifying therapies
FC: Functional connectivity
EAE: Experimental autoimmune encephalomyelitis
GABA: gamma-Aminobutyric acid
GM: Gray matter
LTD: long-term depression
LTP: long-term potentiation
MEP: Motor evoked potentials
MS: Multiple sclerosis
NIBS: Non-invasive brain stimulation
PPMS: Primary progressive MS
PwMS: people living with MS
RIS: radiologically isolated syndrome
RRMS: Relapsing-remitting MS
Rs-FC: resting-state Functional Connectivity
rs-fMRI: resting-state functional MRI
rTMS: repetitive transcranial magnetic stimulation
SC: Social cognition
SPMS: Secondary progressive MS
tDCS: transcranial direct current stimulation.
